# Supplementary material for: The Immunogenicity and Safety of Mycobacterium tuberculosis-mosR-Based Double Deletion Strain in Mice
Source: Microorganisms. 2023 Aug 18;11(8):2105. doi: 10.3390/microorganisms11082105 (PMC10459135; doi:10.3390/microorganisms11082105)
Supplement: Supplementary file 1 [file microorganisms-11-02105-s001.zip › microorganisms-2541640-supplementary.pdf]

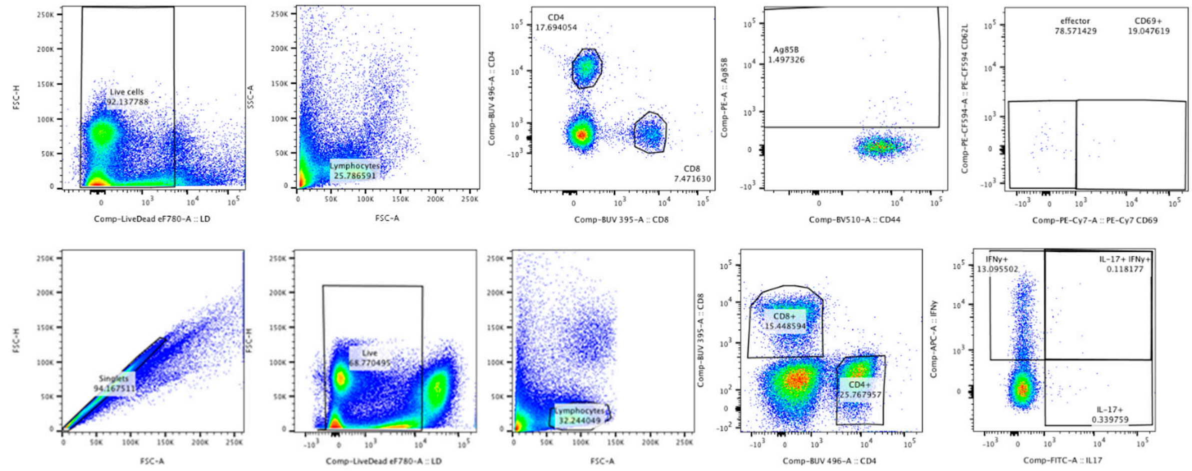

**Supplementary Figure S1.** Examples of Flow Gating Strategy used for Surface stain Samples (top) and intracellular cytokine stains (bottom).

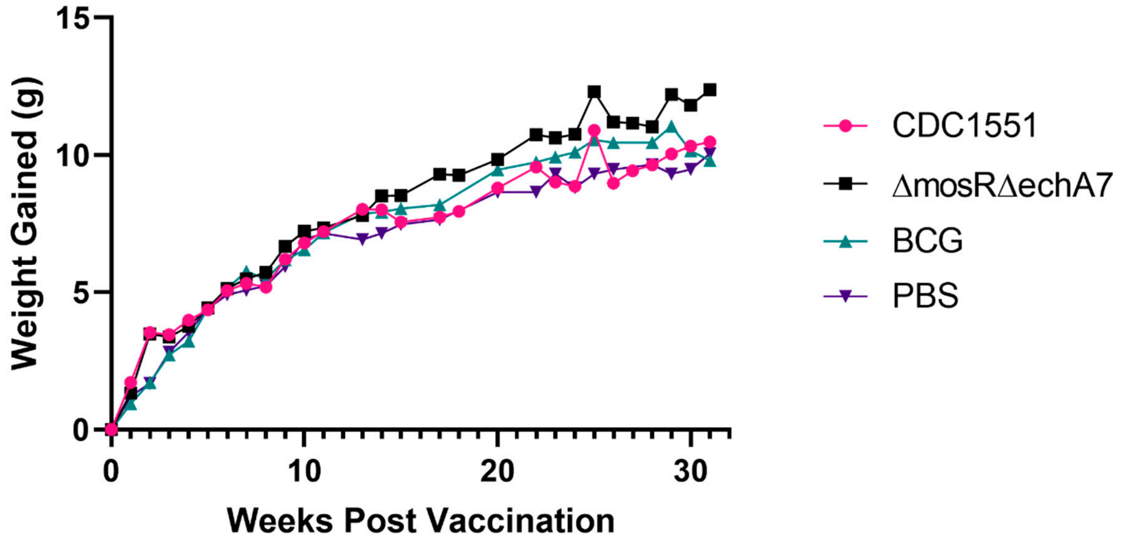


**Supplementary Figure S2.** Average weight gain (g) of mouse groups over 32weeks post-vaccination.
